# Untiring Researches for Alternative Resources of Rhizoma Paridis

**DOI:** 10.1007/s13659-018-0179-5

**Published:** 2018-07-04

**Authors:** Xu-Jie Qin, Wei Ni, Chang-Xiang Chen, Hai-Yang Liu

**Affiliations:** State Key Laboratory of Phytochemistry and Plant Resources in West China, Kunming Institute of Botany, Chinese Academy of Sciences, and Yunnan Key Laboratory of Medicinal Chemistry, Kunming, 650201 People’s Republic of China

**Keywords:** Rhizoma Paridis, *Paris polyphylla*, Alternative resources, Steroidal saponins, Bioactivities

## Abstract

Rhizoma Paridis (RP, 重楼), a traditional Chinese medicine, is the rhizoma of *Paris polyphylla* var. *yunnanensis* (PPY) or *P. polyphylla* var. *chinensis* which are widely used as important raw materials for several Chinese patent drugs. However, the wild resources of these herbs have become less and less due to their slow-growing characteristics and previously excessive excavation. This review covers untiring investigations on alternative resources of RP by our research group over the past decades, including non-medicinal parts of PPY as well as other plants of Liliaceae and Liliflorae families. The arial parts of PPY and the whole plants of *Trillium kamtschaticum* might be alternative resources for RP based on the fact that they shared the same or similar saponins and bioactivities.

## Introduction

The genus *Paris* (Liliaceae) comprises approximately 32 plant species throughout the world and with 26 species found in Southwest China. [1–7]. Among them, the dried rhizoma of *Paris polyphylla* var. *yunnanensis* (PPY) and *P. polyphylla* var. *chinensis* (PPC), both called Rhizoma Paridis (RP) in China, have long been recorded in Chinese Pharmacopoeia as a traditional Chinese medicine to treat furuncle, snakebite, injuries from falls and convulsion, epilepsy, and sore throat [8]. Because of their remarkable medicinal functions, PPY and PPC have been a hot topic within the medicinal chemistry and drug discovery community since the 1970s. Previous studies revealed that PPY and PPC were rich sources of spirostanol (diosgenin and pennogenin) saponins [9–24] responsible for various pharmacological effects, such as cytotoxic and antitumor [13–20], antifungal [21, 22], and haemostatic bioactivities [23, 24]. The available supplies of PPY and PPC are facing increasing shortage based on the fact that their rhizomes can only be harvested until they have grown more than 7 years and the consumption by the pharmaceutical industry of these herbs have increased sharply in recent years. Thus, it is really imperative to search for other saponins or resources that might be substitutes for RP. Over the past 34 years, in order to find valid and alternative resources of RP, our research group have made great effort to phytochemically investigated on the non-medicinal parts of PPY as well as other plants of Liliaceae and Liliflorae families according to their genetic and phylogenetic relationships, which led to the isolation of identical or similar bioactive constituents with those of RP. As a result, a total of 184 saponins and including 120 new ones were obtained and identified, some of which showed interesting bioactive effects as those of RP. This paper mainly describes our untiring researches that can provide active ingredients for alternative resources of RP.

## Steroidal Sapogenins and Saponins

According to the fact that the steroidal saponins are the bioactive constituents of RP, the steroidal sapogenins and saponins of non-medicinal parts of PPY and other *Paris*, *Ypsilandra*, *Trillium*, and *Tacca* plants have been investigated, which led to the isolation of 17 new steroidal sapogenins and 103 steroidal saponins, along with 64 known analogues.

### Non-medicinal Parts of PPY and Other *Paris* Species (Liliaceae)

**Fig. 1:**
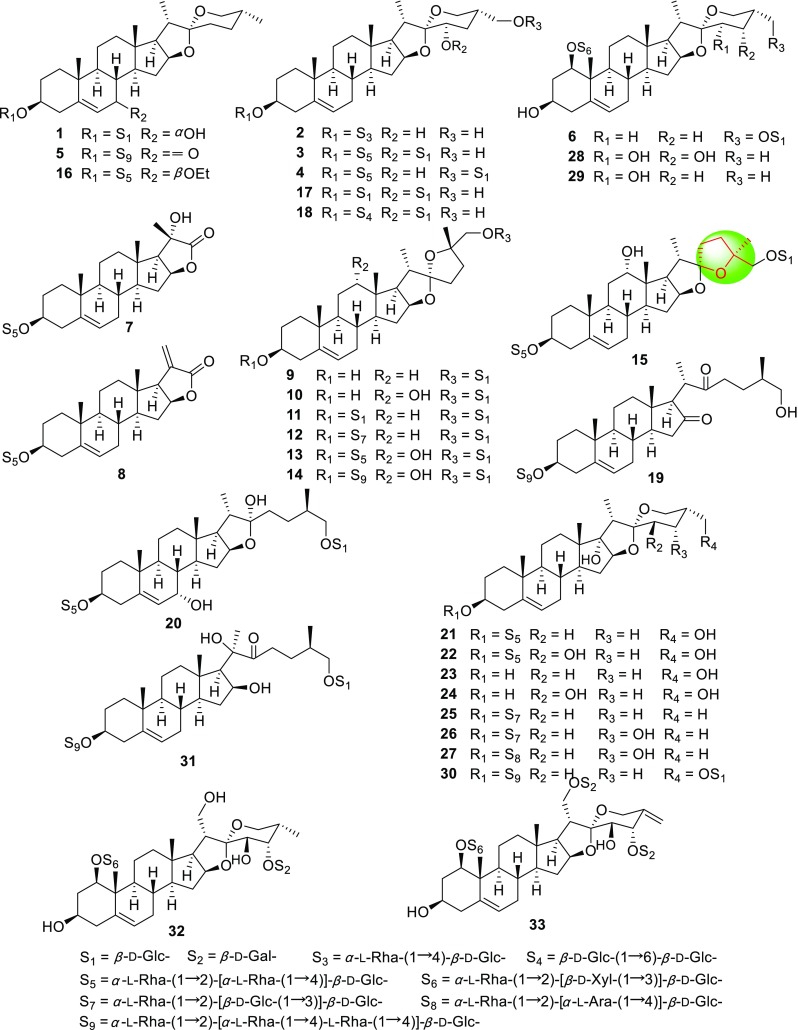
New steroidal sapogenins and saponins from non-medicinal parts of PPY and other *Paris* species

Although the renewable aerial parts of PPY yearly have not been used as medicinal materials, in order to clarify the difference of chemical constituents between medicinal and non-medicinal parts (the stems and leaves) of PPY and to improve the efficiency of resources usage, our systematically phytochemical investigations on the non-medical parts of PPY led to the isolation of 22 new steroidal saponins (Fig. 1; Table 1), named chonglouosides SL-1–SL-20 (**1**–**20**) [25–27], polyphyllosides III (**21**) and IV (**22**) [28], as well as two new steroidal sapogenins, named 27-hydroxylpennogenin (**23**) and 27,23*β*-dihydroxylpennogenin (**24**) [29]. In addition, three new pennogenin saponins (**25**–**27**) [30, 31], three new spirostanol saponins (**28**–**30**) and one new cholestane saponin (**31**) [32], and two new highly oxygenated spirostanol saponins (**32** and **33**) [33] were isolated from *P. axialis* (rhizomes), *P. verticillata* (aerial parts), and *P. polyphylla* var. *stenophylla* (rhizomes), respectively (Fig. 1). It was worth noting that saponins **7** and **8** were C_22_-steroidal lactone saponins which were isolated from genus *Paris* for the first time, while **9**–**15** were rare nuatigenin saponins with a furan ring that firstly obtained from species of Liliaceae family.

**Table 1:** New steroidal sapogenins and saponins from non-medicinal parts of PPY and other *Paris* species

Nos.	Names	Species	Parts	References
**1**	Chonglouoside SL-1	PPY	Stems and leaves	[25]
**2**	Chonglouoside SL-2	PPY	Stems and leaves	[25]
**3**	Chonglouoside SL-3	PPY	Stems and leaves	[25]
**4**	Chonglouoside SL-4	PPY	Stems and leaves	[25]
**5**	Chonglouoside SL-5	PPY	Stems and leaves	[25]
**6**	Chonglouoside SL-6	PPY	Stems and leaves	[25]
**7**	Chonglouoside SL-7	PPY	Stems and leaves	[26]
**8**	Chonglouoside SL-8	PPY	Stems and leaves	[26]
**9**	Chonglouoside SL-9	PPY	Stems and leaves	[27]
**10**	Chonglouoside SL-10	PPY	Stems and leaves	[27]
**11**	Chonglouoside SL-11	PPY	Stems and leaves	[27]
**12**	Chonglouoside SL-12	PPY	Stems and leaves	[27]
**13**	Chonglouoside SL-13	PPY	Stems and leaves	[27]
**14**	Chonglouoside SL-14	PPY	Stems and leaves	[27]
**15**	Chonglouoside SL-15	PPY	Stems and leaves	[27]
**16**	Chonglouoside SL-16	PPY	Stems and leaves	[27]
**17**	Chonglouoside SL-17	PPY	Stems and leaves	[27]
**18**	Chonglouoside SL-18	PPY	Stems and leaves	[27]
**19**	Chonglouoside SL-19	PPY	Stems and leaves	[27]
**20**	Chonglouoside SL-20	PPY	Stems and leaves	[27]
**21**	Polyphylloside III	PPY	Aerial parts	[28]
**22**	Polyphylloside IV	PPY	Aerial parts	[28]
**23**	27-Hydroxyl-pennogenin	PPY	Aerial parts	[29]
**24**	27,23*β*-Dihydroxyl-pennogenin	PPY	Aerial parts	[29]
**25**	Pennogenin-3-*O*-*β*-d-glucopyranosyl-(1 → 3)-[*α*-l-rhamnopyranosyl(1 → 2)]-*β*-d-glucopyranoside	*P. axialis*	Rhizomes	[30]
**26**	24*α*-Hydroxyl-pennogenin-3-*O*-*β*-d-glucopyranosyl-(1 → 3)-[*α*-l-rhamnopyranosyl(1 → 2)]-*β*-d-glucopyranoside	*P. axialis*	Rhizomes	[30]
**27**	24*α*-Hydroxyl-pennogenin-3-*O*-*α*-l-rhamnopyranosyl-(1 → 2)-[*α*-l-arabinofuranosyl(1 → 4)]-*β*-d-glucopyranoside	*P. axialis*	Rhizomes	[31]
**28**	Parisverticoside A	*P. verticillata*	Aerial parts	[32]
**29**	Parisverticoside B	*P. verticillata*	Aerial parts	[32]
**30**	Parisverticoside C	*P. verticillata*	Aerial parts	[32]
**31**	Parisverticoside D	*P. verticillata*	Aerial parts	[32]
**32**	Paristenoside A	*P. polyphylla* var*. stenophylla*	Rhizomes	[33]
**33**	Paristenoside B	*P. polyphylla* var*. stenophylla*	Rhizomes	[33]

### *Ypsilandra* Species (Liliaceae)

**Fig. 2:**
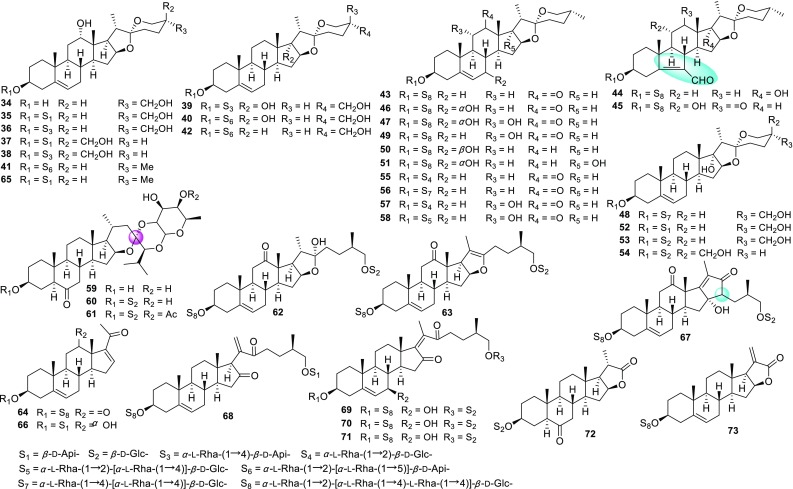
New steroidal sapogenins and saponins from *Ypsilandra* species

*Ypsilandra* (Liliaceae), a small genus including only five species, is widely distributed in Southwest China and Myanmar [34]. We speculate that *Ypsilandra* species should produce similar steroidal derivatives as those of *Paris* due to their genetic and phylogenetic relationships. Although *Y. thibetica* has been used as a folk medicine for treating uterine bleeding and traumatic hemorrhage [35, 36], the chemical constituents of *Ypsilandra* species have not been studied before our investigations. A total of two new sapogenins and 38 saponins (Fig. 2; Table 2) have been reported from the whole plants of *Y. thibetica*, *Y. parviflora*, and *Y. yunnanensis* up to 2017 by our research group, namely, isoypsilandrogenin (**34**), isoypsilandrosides A (**35**) and B (**36**), ypsilandrosides A (**37**) and B (**38**) [37], ypsilandrosides C–G (**39**–**43**) [38], ypsilandrosides H–L (**44**–**48**) [39], ypsilandrosides M–O (**49**–**51**) [40], ypsiparosides A–G (**52**–**58**) [41], ypsilanogenin (**59**), ypsilanogenin 3-*O*-*β*-d-glucopyranoside (**60**), 4′-acetylypsilanogenin 3-*O*-*β*-d-glucopyranoside (**61**) [42], ypsilandrosides P–R (**62**–**64**) [43], ypsilandrosides S (**65**) and T (**66**) [44], ypsiyunnosides A–E (**67**–**71**) [45], and ypsilactosides A (**71**) and B (**72**) [46]. These new saponins were usually the oxygenated derivatives at C-6, C-7, C-11, and C-12 of those known analogues and some of these isolates had unpredicted aglycones. To be more specific, saponins **44** and **45** represented the first example with a novel 5(6 → 7) abeo-steroidal aglycone, whereas **59**–**61** were unusual 23-spirocholestane derivatives and **67** possessed a rare 6/6/6/5/5 fused-rings cholestanol skeleton.

**Table 2:** New steroidal sapogenins and saponins from *Ypsilandra* species (Liliaceae)

Nos.	Names	Species	Parts	References
**34**	Isoypsilandrogenin	*Y. thibetica*	Whole plants	[37]
**35**	Isoypsilandroside A	*Y. thibetica*	Whole plants	[37]
**36**	Isoypsilandroside B	*Y. thibetica*	Whole plants	[37]
**37**	Ypsilandroside A	*Y. thibetica*	Whole plants	[37]
**38**	Ypsilandroside B	*Y. thibetica*	Whole plants	[37]
**39**	Ypsilandroside C	*Y. thibetica*	Whole plants	[38]
**40**	Ypsilandroside D	*Y. thibetica*	Whole plants	[38]
**41**	Ypsilandroside E	*Y. thibetica*	Whole plants	[38]
**42**	Ypsilandroside F	*Y. thibetica*	Whole plants	[38]
**43**	Ypsilandroside G	*Y. thibetica*	Whole plants	[38]
**44**	Ypsilandroside H	*Y. thibetica*	Whole plants	[39]
**45**	Ypsilandroside I	*Y. thibetica*	Whole plants	[39]
**46**	Ypsilandroside J	*Y. thibetica*	Whole plants	[39]
**47**	Ypsilandroside K	*Y. thibetica*	Whole plants	[39]
**48**	Ypsilandroside L	*Y. thibetica*	Whole plants	[39]
**49**	Ypsilandroside M	*Y. thibetica*	Whole plants	[40]
**50**	Ypsilandroside N	*Y. thibetica*	Whole plants	[40]
**51**	Ypsilandroside O	*Y. thibetica*	Whole plants	[40]
**52**	Ypsiparoside A	*Y. parviflora*	Whole plants	[40]
**53**	Ypsiparoside B	*Y. parviflora*	Whole plants	[41]
**54**	Ypsiparoside C	*Y. parviflora*	Whole plants	[41]
**55**	Ypsiparoside D	*Y. parviflora*	Whole plants	[41]
**56**	Ypsiparoside E	*Y. parviflora*	Whole plants	[41]
**57**	Ypsiparoside F	*Y. parviflora*	Whole plants	[41]
**58**	Ypsiparoside G	*Y. parviflora*	Whole plants	[41]
**59**	Ypsilanogenin	*Y. thibetica*	Whole plants	[42]
**60**	Ypsilanogenin 3-*O*-*β*-d-glucopyranoside	*Y. thibetica*	Whole plants	[42]
**61**	4′-Acetylypsilanogenin 3-*O*-*β*-d-glucopyranoside	*Y. thibetica*	Whole plants	[42]
**62**	Ypsilandroside P	*Y. thibetica*	Whole plants	[43]
**63**	Ypsilandroside Q	*Y. thibetica*	Whole plants	[43]
**64**	Ypsilandroside R	*Y. thibetica*	Whole plants	[43]
**65**	Ypsilandroside S	*Y. thibetica*	Whole plants	[44]
**66**	Ypsilandroside T	*Y. thibetica*	Whole plants	[44]
**67**	Ysiyunnoside A	*Y. yunnanensis*	Whole plants	[45]
**68**	Ysiyunnoside B	*Y. yunnanensis*	Whole plants	[45]
**69**	Ysiyunnoside C	*Y. yunnanensis*	Whole plants	[45]
**70**	Ysiyunnoside D	*Y. yunnanensis*	Whole plants	[45]
**71**	Ysiyunnoside E	*Y. yunnanensis*	Whole plants	[45]
**72**	Ypsilactoside A	*Y. thibetica*	Whole plants	[46]
**73**	Ypsilactoside B	*Y. thibetica*	Whole plants	[46]

### *Trillium* Species (Liliaceae)

**Fig. 3:**
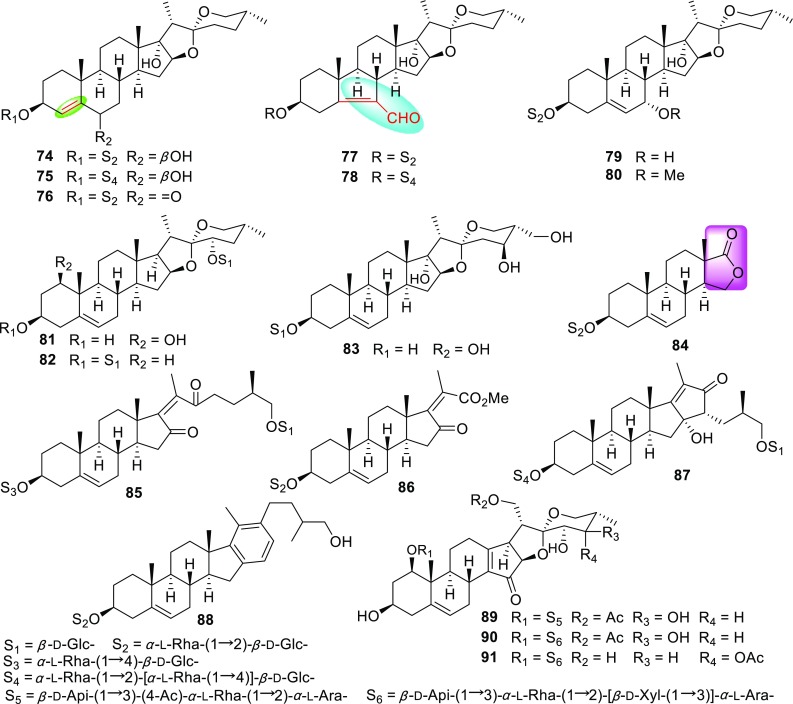
New steroidal saponins from *Trillium* species

The *Trillium* genus consists of approximately 49 species throughout the world. However, only three species, *T. kamtschaticum*, *T. tschonoskii*, and *T. govanianum*, are found in Hubei, Sichuan, Yunnan, and Xizang Provinces of China. The rhizomes of *T. kamtschaticum*, called “Toudingyikezhu” in Chinese, have been traditionally use by Chinese minorities (Tujia and Miao people) for the treatment of traumatic hemorrhage [47, 48]. In addition, some pennogenin saponins have been reported from *Trillium* species [49, 50] and the crude extract of the whole plants of *T. kamtschaticum* displayed significant induced-platelet aggregation activity at a concentration of 1.5 mg/mL as revealed by our initiatory test. All these information strongly inspired us to investigated the hemostatic constituents of the whole plats of *T. kamtschaticum*, resulting in the isolation of 18 new steroidal saponins (Fig. 3; Table 3), named trillikamtosides A–R (**74**–**91**) [51, 52]. Interestingly, some of them were determined to have rare aglycone moieties. For instance, the aglycones of **73**–**75** had unique 3*β*,17*α*-dihydroxyspirostanes featuring a double bond between C-4 and C-5, **7**6 and **77** represented a rare class of spirostanol saponins which possess a 5(6–7) abeo-steroidal aglycone, and **83** possessed a rare aglycone with a 16-oxaandrost-5-en-3-ol-17-one moiety. Moreover, saponins **84** and **86** were schizolytic derivatives of those furanstanols and **89**–**91** were new trillenogenin saponins being only found in *Trillium* plants. The relevant researches of the othe*r Trillium* specie*s* are going on in our laboratory.

**Table 3:** New steroidal saponins from the whole plants of *T. kamtschaticum*

No.	Name	Species	References
**74**	Trillikamtoside A	*T. kamtschaticum*	[51]
**75**	Trillikamtoside B	*T. kamtschaticum*	[51]
**76**	Trillikamtoside C	*T. kamtschaticum*	[51]
**77**	Trillikamtoside D	*T. kamtschaticum*	[51]
**78**	Trillikamtoside E	*T. kamtschaticum*	[51]
**79**	Trillikamtoside F	*T. kamtschaticum*	[51]
**80**	Trillikamtoside G	*T. kamtschaticum*	[51]
**81**	Trillikamtoside H	*T. kamtschaticum*	[51]
**82**	Trillikamtoside I	*T. kamtschaticum*	[51]
**83**	Trillikamtoside J	*T. kamtschaticum*	[51]
**84**	Trillikamtoside K	*T. kamtschaticum*	[52]
**85**	Trillikamtoside L	*T. kamtschaticum*	[52]
**86**	Trillikamtoside M	*T. kamtschaticum*	[52]
**87**	Trillikamtoside N	*T. kamtschaticum*	[52]
**88**	Trillikamtoside O	*T. kamtschaticum*	[52]
**89**	Trillikamtoside P	*T. kamtschaticum*	[52]
**90**	Trillikamtoside Q	*T. kamtschaticum*	[52]
**91**	Trillikamtoside R	*T. kamtschaticum*	[52]

### *Tacca* Species (Taccaceae)

**Fig. 4:**
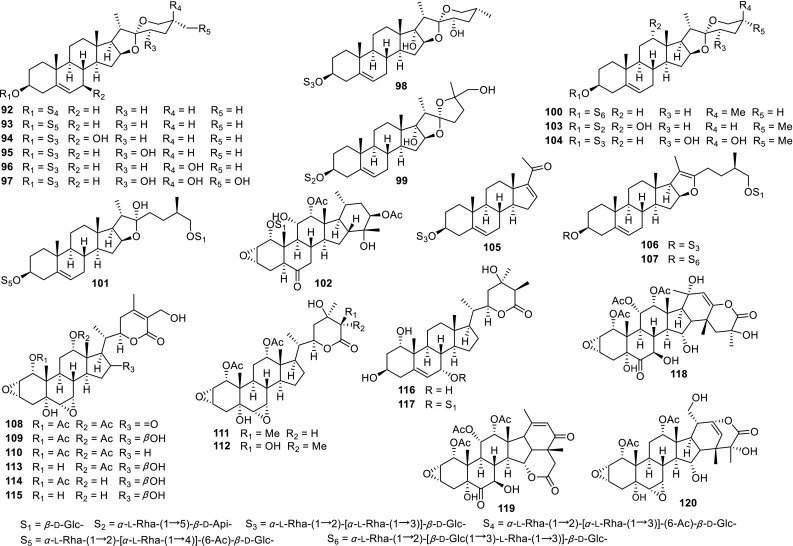
New steroidal sapogenins and saponins from *Tacca* species

Compared with the genera of Liliaceae family, the *Tacca* plants are very limited. In order to discuss/explore whether the *Tacca* species possess the same steroidal constituents as that of RP, our group investigated the phytochemicals of two *Tacca* species (*T. plantaginea and T. subflabellata*). The results led to the structural characterization of eight new spirostane saponins, named taccaosides E–L (**92**–**99**) [53], taccaoside C (**100**) [54], taccasubosides B (**103**) and C (**104**) [55], three furostanol saponins, named taccaoside D (**101**) [54], taccaosides A (**106**) and B (**107**) [56], a new C_21_ steroidal saponin, taccasuboside D (**105**) [55], and 13 new withanolides, named taccasuboside A (**102**) [55], plantagiolides A–E (**108**–**112**) [57], plantagiolide F (**113**) [58], plantagiolides K–N (**114**–**117**) [59], and taccalonolides W–Y (**118**–**120**) [60] (Fig. 4; Table 4). Although withanolides **108**–**117** and taccalonolides **118**–**120** were also steroidal derivatives with 28 carbons, they may be the taxonomic markers of *Tacca* species.

**Table 4:** New steroidal sapogenins and saponins from *Tacca* species

Nos.	Names	Species	Parts	References
**92**	Taccaoside E	*T. plantaginea*	Whole plants	[53]
**93**	Taccaoside F	*T. plantaginea*	Whole plants	[53]
**94**	Taccaoside G	*T. plantaginea*	Whole plants	[53]
**95**	Taccaoside H	*T. plantaginea*	Whole plants	[53]
**96**	Taccaoside I	*T. plantaginea*	Whole plants	[53]
**97**	Taccaoside J	*T. plantaginea*	Whole plants	[53]
**98**	Taccaoside K	*T. plantaginea*	Whole plants	[53]
**99**	Taccaoside L	*T. plantaginea*	Whole plants	[53]
**100**	Taccaoside C	*T. plantaginea*	Whole plants	[54]
**101**	Taccaoside D	*T. plantaginea*	Whole plants	[54]
**102**	Taccasuboside A	*T. subflabellata*	Whole plants	[55]
**103**	Taccasuboside B	*T. subflabellata*	Whole plants	[55]
**104**	Taccasuboside C	*T. subflabellata*	Whole plants	[55]
**105**	Taccasuboside D	*T. subflabellata*	Whole plants	[55]
**106**	Taccaoside A	*T. plantaginea*	Rhizomes	[56]
**107**	Taccaoside B	*T. plantaginea*	Rhizomes	[56]
**108**	Plantagiolide A	*T. plantaginea*	Whole plants	[57]
**109**	Plantagiolide B	*T. plantaginea*	Whole plants	[57]
**110**	Plantagiolide C	*T. plantaginea*	Whole plants	[57]
**111**	Plantagiolide D	*T. plantaginea*	Whole plants	[57]
**112**	Plantagiolide E	*T. plantaginea*	Whole plants	[57]
**113**	Plantagiolide F	*T. plantaginea*	Whole plants	[58]
**114**	Plantagiolide K	*T. plantaginea*	Whole plants	[59]
**115**	Plantagiolide L	*T. plantaginea*	Whole plants	[59]
**116**	Plantagiolide M	*T. plantaginea*	Whole plants	[59]
**117**	Plantagiolide N	*T. plantaginea*	Whole plants	[59]
**118**	Taccalonolide W	*T. plantaginea*	Whole plants	[60]
**119**	Taccalonolide X	*T. plantaginea*	Whole plants	[60]
**120**	Taccalonolide Y	*T. plantaginea*	Whole plants	[60]

### Known Sapogenin and Saponins Obtained from the Non-medicinal Parts of PPY and Other *Paris*, *Ypsilandra*, *Trillium*, and *Tacca* Plants

**Fig. 5:**
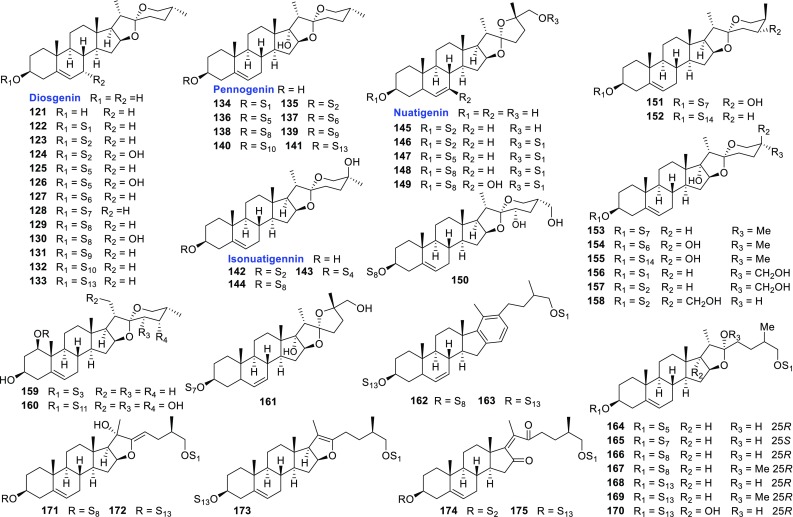

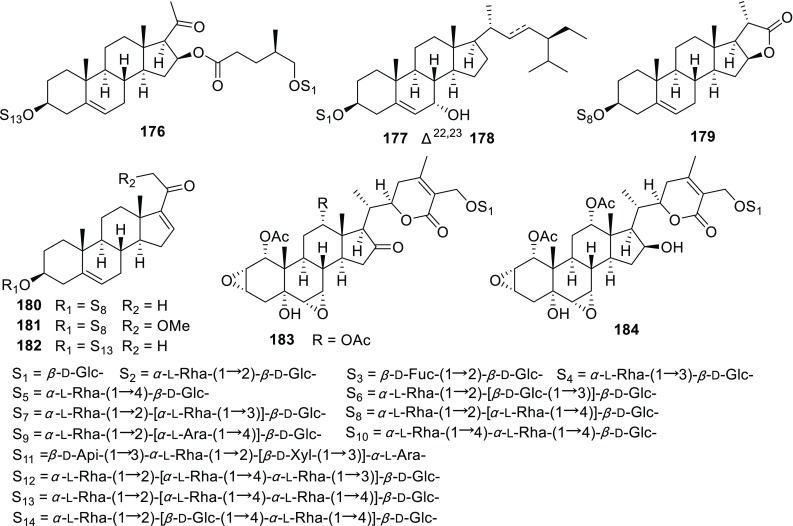
Known steroidal sapogenins and saponins

Apart from the above mentioned new saponins, 1 known sapogenin and 63 known saponins were also identified from the aforementioned species (Fig. 5; Table 5). Compared with those new isolates, these known compounds usually shared the aglycones with lower oxidation degrees.

**Table 5:** Known steroidal sapogenins and saponins

Nos.	Names	Species	Parts	References
**121**	Diosgenin	PPY	Stems and leaves	[25]
**122**	Polyphyllin A	PPY	Stems and leaves	[25]
**123**	Paris saponin V	PPY	Stems and leaves	[25]
		*P. axialis*	rhizomes	[31]
		*P. delavayi*	Rhizomes	[31]
		*Y. thibetica*	Whole plants	[38]
**124**	Sansevierin A	PPY	Stems and leaves	[25]
**125**	Progenin II	PPY	Stems and leaves	[25]
**126**	Disoseptemloside D	PPY	Stems and leaves	[25]
**127**	Diosgenin-3-*O*-*β*-d-glucopyranosyl-(1 → 3)-[*α*-l-rhamnopyranosyl(1 → 2)]-*β*-d-glucopyranoside	*P. axialis*	Rhizomes	[30]
**128**	Taccaoside	*T. plantaginea*	Whole plants	[53]
		*T. chanteraeri*	Rhizomes	[66]
**129**	Dioscin	PPY	Stems and leaves	[25]
		*Y. thibetica*	Whole plants	[38]
		*T. plantaginea*	Whole plants	[53]
**130**	Disoseptemloside E	PPY	Stems and leaves	[25]
**131**	Paris saponin I	*P. axialis*	Rhizomes	[31]
		*P. delavayi*	Rhizomes	[31]
		*P. dunniana*	Rhizomes	[31]
		*P. luquanensis*	Rhizomes	[62]
**132**	Diosgenin-3-*O*-*α*-l-rhamnopyranosyl-(1 → 4)-*α*-l-rhamnopyranosyl(1 → 4)-*β*-d-glucopyranoside	PPY	Stems and leaves	[27]
		*P. verticillata*	Aerial parts	[32]
		*Y. thibetica*	Whole plants	[38]
**133**	Paris saponin II	PPY	Stems and leaves	[25]
		*Y. thibetica*	Whole plants	[38]
		*Y. parviflora*	Whole plants	[41]
**134**	Pennogenin 3-*O*-*β*-d-glucopyranoside	*T. kamtschaticum*	Whole plants	[51]
**135**	Paris saponin VI	PPY	Stems and leaves	[25]
		*P. axialis*	Rhizomes	[31]
		*P. delavayi*	Rhizomes	[31]
		*T. kamtschaticum*	Whole plants	[51]
**136**	Floribundasaponin B	*Y. thibetica*	Whole plants	[38]
		*T. kamtschaticum*	Whole plants	[51]
**137**	Pennogenin 3-*O*-*β*-d-glucopyranosyl-(1 → 3)-[*α*-l-rhamnopyranosyl(1 → 2)]-*β*-d-glucopyranoside	*P. axialis*	Rhizomes	[31]
**138**	Pennogenin 3-*O*-*β*-chacotrioside	PPY	Aerial parts	[64]
		*T. kamtschaticum*	Whole plants	[51]
		*Y. thibetica*	Whole plants	[38]
		*Y. parviflora*	Whole plants	[41]
**139**	Paris saponin H	*P. axialis*	Rhizomes	[31]
		*P. delavayi*	Rhizomes	[31]
		*P. dunniana*	Rhizomes	[31]
		*P. luquanensis*	Rhizomes	[62]
**140**	Pennogenin 3-*O*-*α*-l-rhamnopyranosyl-(1 → 4)-*α*-l-rhamnopyranosyl-(1 → 4)-*β*-d-glucopyranoside	PPY	Stems and leaves	[25]
		*P. verticillata*	Aerial parts	[32]
		*T. kamtschaticum*	Whole plants	[51]
		*Y. parviflora*	Whole plants	[48]
**141**	Paris saponin VII	PPY	Stems and leaves	[25]
		*P. verticillata*	Aerial parts	[32]
		*P. luquanensis*	Rhizomes	[62]
		PPY	Seeds	[63]
		*T. kamtschaticum*	Whole plants	[51]
		*Y. parviflora*	Whole plants	[41]
		*Y. thibetica*	Whole plants	[39]
**142**	Isonuatigenin 3-*O*-*α*-l-rhamnopyranosyl-(1–2)-*β*-d-glucopyranoside	PPY	Stems and leaves	[25]
**143**	Disoseptemloside H	PPY	Stems and leaves	[25]
**144**	Pennogenin 3-*O*-*α*-l-rhamnopyranosyl-(1 → 2)-[*α*-l-rhamnopyranosyl(1 → 4)]-*β*-d-glucopyranoside	PPY	Aerial parts	[65]
**145**	Nuatigenin 3-*O*-*α*-l-rhamnopyranosyl-(1 → 2)-*β*-d-glucopyranoside	PPY	Stems and leaves	[27]
**146**	26-*O*-*β*-d-glucopyranosyl nuatigenin 3-*O*-*α*-l-rhamnopyranosyl-(1 → 2)-*β*-d-glucopyranoside	PPY	Stems and leaves	[27]
**147**	26-*O*-*β*-d-glucopyranosyl nuatigenin 3-*O*-*α*-l-rhamnopyranosyl-(1 → 4)-*β*-d-glucopyranoside	PPY	Stems and leaves	[27]
**148**	26-*O*-*β*-d-glucopyranosyl nuatigenin 3-*O*-*α*-l-rhamnopyranosyl-(1 → 2)-[*α*-l-rhamnopyranosyl-(1 → 4)]-*β*-d-glucopyranoside	PPY	Aerial parts	[65]
**149**	Abutiloside L	PPY	Stems and leaves	[27]
**150**	Borassoside B	PPY	Stems and leaves	[27]
**151**	(24*S*,25*R*)-spirost-5-en-3*β*,24-diol-3-*O*-*α*-l-rhamnopyranosyl-(1 → 2)-[*α*-l-rhamnopyranosyl(1 → 3)]-*β*-d-glucopyranoside	*T. plantaginea*	Whole plants	[53]
**152**	(25*S*)-spirost-5-en-3*β*-ol-3-*O*-*α*-l-rhamnopyranosyl-(1 → 2)-[*β*-d-glucopyranosyl-(1 → 4)-*α*-l-rhamnopyranosyl-(1 → 3)]-*β*-d-glucopyranoside	*T. plantaginea*	Whole plants	[53]
**153**	Spiroconazole A	*T. plantaginea*	Whole plants	[53]
**154**	Diosbulbiside A	*T. plantaginea*	Whole plants	[53]
**155**	Diosbulbiside B	*T. plantaginea*	Whole plants	[53]
**156**	(25*S*)-27-hydroxypennogenin 3-*O*-*β*-d-glucopyranoside	*T. kamtschaticum*	Whole plants	[51]
**157**	(25*S*)-27-hydroxypennogenin-3-*O*-*α*-l-rhamnopyranosyl-(1 → 2)-*β*-d-glucopyranoside	*T. kamtschaticum*	Whole plants	[51]
**158**	Trikamsteroside A	*T. kamtschaticum*	Whole plants	[51]
**159**	Ophiopogonin B	*T. kamtschaticum*	Whole plants	[51]
**160**	Trikamsteroside E	*T. kamtschaticum*	Whole plants	[52]
**161**	Diosbulbiside E	*T. plantaginea*	Whole plants	[53]
**162**	Aethioside A	*T. kamtschaticum*	Whole plants	[52]
**163**	Parispseudoside A	*P. verticillata*	Aerial parts	[32]
		*Y. parviflora*	Whole plants	[41]
		*Y. thibetica*	Whole plants	[43]
**164**	Protoprogenin II	*Y. thibetica*	Whole plants	[43]
**165**	26-*O*-*β*-d-glucopyranosyl-(25*S*)-3*β*,22ξ,26-triol-furost-5-ene 3-*O*-*α*-l-rhamnopyranosyl-(1 → 2) -[*α*-l-rhamnopyranosyl(1 → 3)]-*β*-d-glucopyranoside	*T. plantaginea*	Whole plants	[54]
		*T. subflabellata*	Whole plants	[55]
**166**	Proto-dioscin	PPY	Stems and leaves	[25]
**167**	Methylprotodioscin	PPY	Stems and leaves	[25]
**168**	Proto-paris saponin II	*P. verticillata*	Aerial parts	[32]
		*Y. parviflora*	Whole plants	[41]
		*Y. thibetica*	Whole plants	[43]
**169**	26-*O*-*β*-d-glucopyranosyl-22-methoxy-3*β*,26-dihydroxy-(25*R*)-furost-5-en-3-*O*-*α*-l-rhamnopyranosyl-(1 → 2)-[*α*-l-rhamnopyranosyl -(1 → 4)-*α*-l-rhamnopyranosyl-(1 → 3)]-*β*-d-glucopyranoside	*P. verticillata*	Aerial parts	[32]
**170**	Proto-paris saponin VII	*P. verticillata*	Aerial parts	[32]
		*Y. thibetica*	Whole plants	[43]
**171**	26-*O*-*β*-d-glucopyranosyl-3*β*,20*α*,26-triol-(25*R*)-5,22-dienofurostan 3-*O*-*α*-l-rhamnopyranosyl-(1 → 2)-[*α*-l-rhamnopyranosyl(1 → 4)]-*β*-d-glucopyranoside	PPY	Stems and leaves	[25]
**172**	Smilaxchinoside B	*P. verticillata*	Aerial parts	[32]
		*Y. thibetica*	Whole plants	[43]
**173**	26-*O*-*β*-d-glucopyranosyl-17(20)-dehydrokryptogenin-3-*O*-*α*-l-rhamnopyranosyl-(1 → 2)-*β*-d-glucopyranoside	*T. kamtschaticum*	Whole plants	[52]
**174**	Pseudoproto Pb	*P. verticillata*	Aerial parts	[32]
		*Y. parviflora*	Whole plants	[41]
		*Y. thibetica*	Whole plants	[43]
**175**	Parispseudoside C	*P. verticillata*	Aerial parts	[32]
		*Y. thibetica*	Whole plants	[43]
		*Y. yunnanensis*	Whole plants	[45]
**176**	26-*O*-*β*-d-glucopyranosyl-3*β*,26-dihydroxy-20,22-*seco*-25(*R*)-furost-5-en-20,22-dione 3-*O*-*α*-l-rhamnopyranosyl-(1 → 4)-*α*-l-rhamnopyranosyl-(1 → 4)-[*α*-l-rhamnopyranosyl-(1 → 2)]-*β*-d-glucopyranoside	*Y. thibetica*	Whole plants	[43]
**177**	7*α*-Hydroxystigmasterol-3-*O*-*β*-d-glucopyranoside	PPY	Stems and leaves	[27]
**178**	7*α*-Hydroxysitosterol-3-*O*-*β*-d-glucopyranoside	PPY	Stems and leaves	[27]
**179**	Dumoside	PPY	Stems and leaves	[26]
**180**	Hypoglaucin H	PPY	Stems and leaves	[25]
		PPY	Aerial parts	[64]
**181**	21-Methoxyl pregna-5,16-dien-3*β*-ol-20-one 3-*O*-*α*-l-rhamnopyranosyl-(1 → 2)-[*α*-l-rhamnopyranosyl(1 → 4)]-*β*-d-glucopyranoside	PPY	Stems and leaves	[25]
**182**	Pregna-5,16-dien-3*β*-ol-20-one 3-*O*-*α*-l-rhamnopyranosyl-(1 → 2)-[*α*-l-rhamnopyranosyl(1 → 4)-*α*-l-rhamnopyranosyl(1 → 4)]-*β*-d-glucopyranoside	*P. verticillata*	Aerial parts	[32]
		*Y. thibetica*	Whole plants	[43]
		PPY	Aerial parts	[64]
**183**	Chantriolide A	*T. subflabellata*	Whole plants	[55]
		*T. plantaginea*	Whole plants	[57]
**184**	Chantriolide B	*T. subflabellata*	Whole plants	[55]

## Bioactivities

Based on the fact that RP is traditionally used as hemostatic, antimicrobial, and antitumor agents, the hemostatic, antimicrobial, and cytotoxic activities of obtained compounds were evaluated to initially confirm that whether the plants could be alternative resources of RP. Our studies revealed that most of the bioactive compounds were spirostanol saponins with only one sugar chain at OH-3.

### Hemostatic Effect

Both the total steroidal saponin moieties and purified saponins of PPY and *T. kamtschaticum* exhibited hemostatic effects. The 70% EtOH eluted fraction of *T. kamtschaticum* crude extract obtained from a macroporous resin column showed 76% maximal platelet aggregation rate at a concentration of 1.5 mg/mL [51]. Subsequently, three pennogenin-type saponins, paris saponin VI (**135**), pennogenin 3-*O*-*β*-chacotrioside (**138**), and paris saponin VII (**141**) were obtained and further proved to display maximal induced platelet aggregation rates (MPARs) of 72, 71, and 62% with EC_50_ values of 0.49, 0.20, and 0.11 mM, respectively [51]. The results also suggested that the hydroxy group at C-17 in pennogenin saponins was indispensable for their hemostatic effects, whereas the introduction of different functional groups in the A, B, or F-ring of pennogenin glycosides could make the hemostatic effect weak or disappear. Interestingly, the total saponin moieties from the above-ground parts and the rhizomes of PPY showed equivalent maximal platelet aggregation rates of 45 and 43% at a concentration of 1.5 mg/mL, respectively [61]. This indicated that the above-ground parts can be an alternative and more sustainable sources for RP. Additionally, two diosgenin-type saponins, ypsilandroside M (**49**), ypsiparoside C (**54**), and paris saponin II (**133**) isolated from *Y. parviflora*, exhibited MPARs of 43, 44 and 55% at the concentration of 0.3 mg/mL, respectively [41]. This indicated that the carbonyl group at C-12 or the sole *α*-l-rhamnopyranosyl-(1 → 4)-*α*-l-rhamnopyranosyl-(1 → 4)-[*α*-l-rhamnopyranosyl-(1 → 2)]-*β*-d-glucopyranosyl moiety at OH-3 was essential for the hemostatic effect of diosgenin saponins.

### Cytotoxic Effect

A number of saponins were proved to have cytotoxicity against various human tumor cells. Two Trillium saponis with a double bond between C-13 and 14 isolated from *T. kamtschaticum*, trillikamtosides P (**89**) and R (**91**), showed cytotoxic effect against HCT116 (colorectal carcinoma) cells with the MIC values of 4.92 and 5.84 μM, respectively [52]. Ypsilandroside G (**43**) obtained from *Y. thibetica* displayed cytotoxic effect against K562 (leukemia) cells with an MIC value of 4.7 μM, and paris saponin VII (**141**) identified from the same species was cytotoxic towards SPC-A-1 (lung carcinoma) and BGC-823 (gastric carcinoma) with the IC_50_ values of 2.6 and 4.0 μM, respectively [38]. Nuatigenin 3-*O*-*α*-l-rhamnopyranosyl-(1 → 2)-*β*-d-glucopyranoside isolated from the stems and leaves of PPY exhibited cytotoxicity against HepG2 (hepatoma) and HEK293 (renal carcinoma) cell lines with IC_50_ values of 2.9 and 5.0 μM, respectively [27]. Taccaoside (**128**), a saponin obtained from *T. plantaginea*, exhibited significant cytotoxicity against HepG2 and HEK293 cell lines with IC_50_ values of 1.2 and 1.7 μM, respectively [53]. Compared with the positive control drug cisplatin (DDP), a furostanol saponin isolated from *T. subflabellata*, 26-*O*-*β*-d-glucopyranosyl-(25*S*)-3*β*,22ξ,26-triol-furost-5-ene 3-*O*-*α*-l-rhamnopyranosyl(1 → 2)-[*α*-l-rhamnopyranosyl(1 → 3)]-*β*-d-glucopyranoside (**165**) showed significant cytotoxicity against HL-60 (leukemic), SMMC-7721 (hepatoma), A549 (lung carcinoma), MCF-7 (breast carcinoma), and SW480 (colon carcinoma) cells with the IC_50_ values of 4.63, 4.34, 3.00, 11.13, and 2.68 μM, respectively [55]. Ypsilandroside P (**62**), a furostanol saponin obtained from *Y. thibetica*, showed inhibition ratio of 86.4 and 75.9% to A549 and HL-60 cells at the concentration of 10.0 μM, respectively [43]. Moreover, the total saponin moieties from the both rhizomes and above-ground parts of PPY showed cytotoxicities against HL-60, A549, SMMC-7721, MCF-7, and SW480 cells [61]. To be more specific, the former displayed cytotoxicities against above-mentioned cancer cells with IC_50_ values of 1.77, 1.75, 5.23, 6.62, and 3.49 μM, whereas the latter was less cytotoxic with IC_50_ values of 9.54, 9.30, 12.61, 8.12, and 11.25 μM, respectively.

### Antimicrobial Effect

Ypsilandroside G (**43**) obtained from *Y. thibetica* showed moderate inhibitory effect on *Candida albicans* with an MIC value of 10 μg/mL [38]. Compared with that of fluconazole (MIC = 52.3 μM), five saponins isolated from *T. kamtschaticum*, named paris saponin VI (**135**), floribundasaponin B (**136**), pennogenin 3-*O*-*β*-chacotrioside (**138**), paris saponin V (**123**), and ophiopogonin B (**159**), displayed significant antifungal activity against *C. albicans* with the MIC values of 21.1, 10.6, 8.8, 21.6, and 11.0 μM, respectively [51]. Chonglouoside SL-6 (**6**), progenin II (**125**), and dumoside (**179**), three steroidal saponins isolated from the stems and leaves, exhibited good antibacterial activity with the MIC values of 3.9, 7.8, and 3.9 μg/mL, respectively [25, 26]. All three spirostanol saponins identified from PPY, paris saponin V (**123**), dioscin (**129**), and paris saponin II (**133**), were revealed to show significant antifungal activities against *C. albicans* 5314 and *C. albicans* Y0109 with an MIC value of 1.95 μg/mL [61]. Also, the total saponin moieties from both the above-ground parts and the rhizomes of PPY exhibited remarkable antifungal activities against *C. albicans* Y0109 with MIC values of 10.3 and 5.15 μg/mL, respectively, compared with that the positive control voriconazole (MIC = 15.63 μg/mL) [61].

## Conclusion

In summary, our continuous effort to search for alternative resources of RP led to the isolation of 184 steroidal derivatives, including 120 new ones. More importantly, several compounds of them displayed remarkable hemostatic, cytotoxic, and antimicrobial effects. Our studies disclosed that the non-medicinal parts of PPY, as well as other plants of *Paris*, *Ypsilandra*, *Trillium*, and Taccaceae family are also resources rich of steroidal saponins similar to those of RP, especially those recorded in Chinese Pharmacopoeia, namely, paris saponins I (**131**), II (**133**), VI (**135**), and VII (**141**). However, the investigations on the total content of these saponins, the related bioactivities of total saponin moieties of the studied species compared with those of RP, and their security capability are quite indispensable to confirm that whether the non-medicinal parts of PPY and other species from *Paris*, *Ypsilandra*, and *Tacca* genera could be safe and dependable alternative resources of RP. The arial parts of PPY and the whole plants of *T. kamtschaticum* might be alternative resources for RP based on the fact that they shared the same or similar saponins and bioactivities. The continuous studies on the saponin constituents of non-medicinal parts of RP and other plants will be carried out in our laboratory which may led to the discovery of more alternative resources for RP.
